# Supplementary material for: Evolutionary genomics of Leishmania braziliensis across the neotropical realm
Source: Commun Biol. 2024 Nov 28;7:1587. doi: 10.1038/s42003-024-07278-z (PMC11605123; doi:10.1038/s42003-024-07278-z)
Supplement: Supplementary file 1 — Supplementary Information [file 42003_2024_7278_MOESM1_ESM.pdf]

Supplementary information for:

Evolutionary genomics of *Leishmania braziliensis* across the Neotropical realm

Senne Heeren*, Mandy Sanders, Jeffrey Jon Shaw, Sinval Pinto Brandão-Filho, Mariana Côrtes Boité, Lilian Motta Cantanhêde, Khaled Chourabi, Ilse Maes, Alejandro Llanos-Cuentas, Jorge Arevalo, Jorge D. Marco, Philippe Lemey, James A. Cotton, Jean-Claude Dujardin, Elisa Cupolillo*, Frederik Van den Broeck*

*Corresponding authors:
Senne Heeren (sheeren@itg.be)
Frederik Van den Broeck (fvandenbroeck@gmail.com)
Elisa Cupolillo (elisa.cupolillo@ioc.fiocruz.br)

Supplementary results

Genomic evidence of interspecific hybridization in *Leishmania* (*Viannia*) parasites

The phylogenetic network further revealed an uncertain reticulated ancestry for six *Leishmania* genomes (LC2484A1, PER182A1, Venez_RA, Venez_PM_H32, OLO1A1 and M4147) (Figure 1c). The distribution of alternate allele read depth frequencies (ARDF) was centered around 0.5 for all six genomes (Supplementary Figure 10), as expected for diploid organisms (both alleles are represented equally). Inspecting the chromosomal ARDF, all putative hybrid isolates revealed to have several genomic regions (or entire chromosomes) that were either largely homozygous or SNP poor amidst highly heterozygous regions (Figure 1b). Generating phylogenetic networks based on the SNPs present in these non-heterozygous regions revealed patterns of i) interspecific hybridization between the *L. braziliensis* and *L. guyanensis* species complexes (LC2484A1, Venez_RA, Venez-PM_H32) (Supplementary Figure 11); and ii) hybridization within the *L. guyanensis* species complex between *L. shawi* and *L. guyanensis*/*L. panemensis* (OLO1A1, M4147) (Supplementary Figure 12). No clear indications explaining the high heterozygosity/mixed ancestry of PER182A1 were found. These patterns of inter- and intraspecific hybridization were further substantiated by PCA-based ancestry estimation (PCAdmix) (Supplementary Figure 13) showing more central positions of the hybrid in contrast to their putative parental species.

Supplementary figures

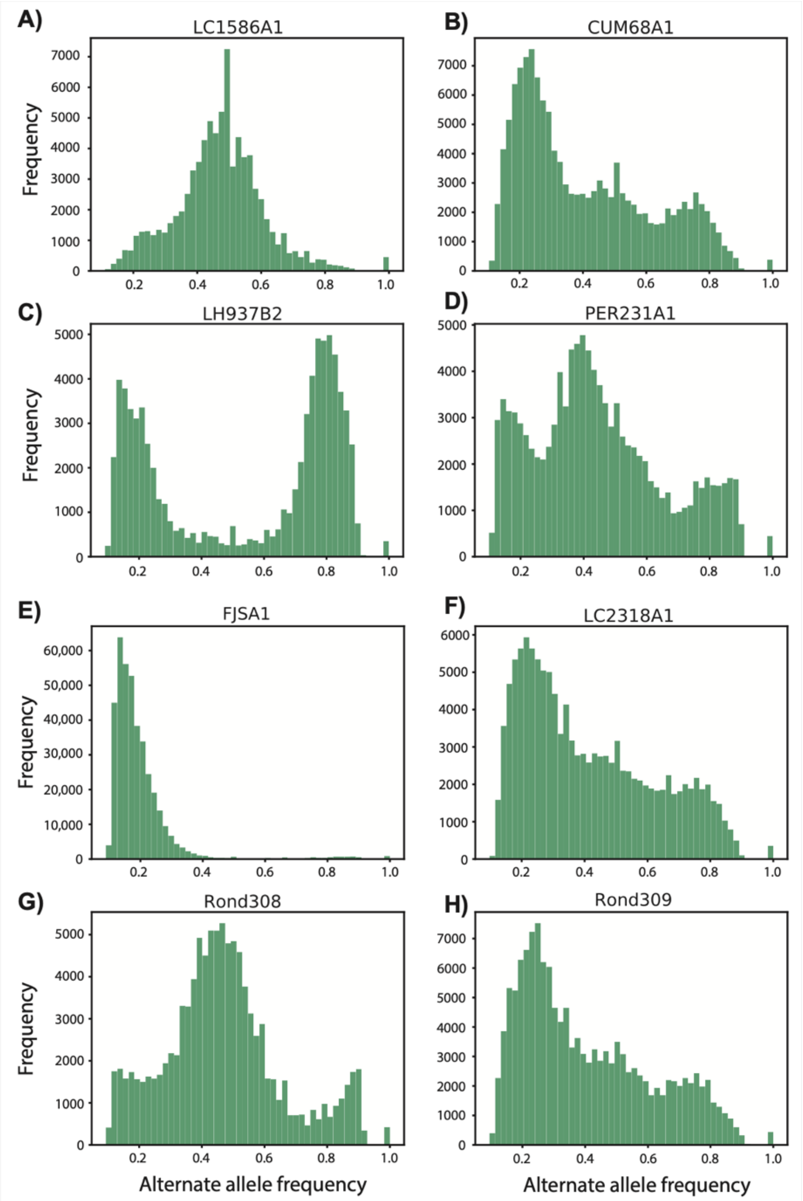

Supplementary Figure 1 Removal of isolates based on alternate allele frequency distributions. Assuming diploidy is the ancestral state within the *L.* (*Viannia*) subgenus (1), the genome-wide distribution of alternate allele frequencies at heterozygous sites should approximate 0.5. Visual inspection of frequency plots confirmed a unimodal distribution centered around 0.5 for 244 isolates. This is exemplified by isolate LC1586A1 **(A)**. Seven isolates were removed for downstream analysis because they showed vastly different distributions **(B-H)** which might be the result of mixed infections.

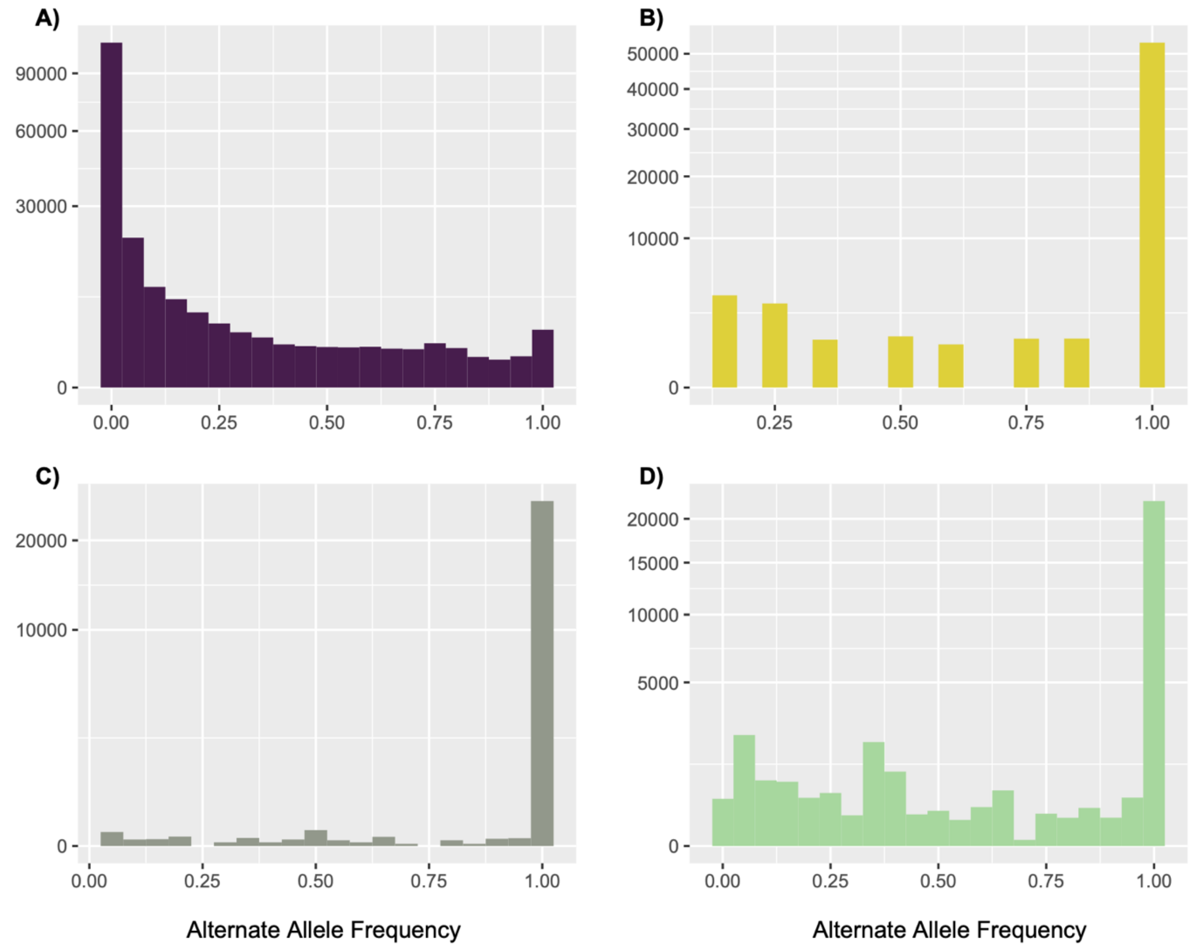

Supplementary Figure 2 Population allele frequency spectra for *L. braziliensis* ecotypes L1 **(A)**, L2 **(B)**, L3 **(C)** and *L. peruviana* **(D)**.

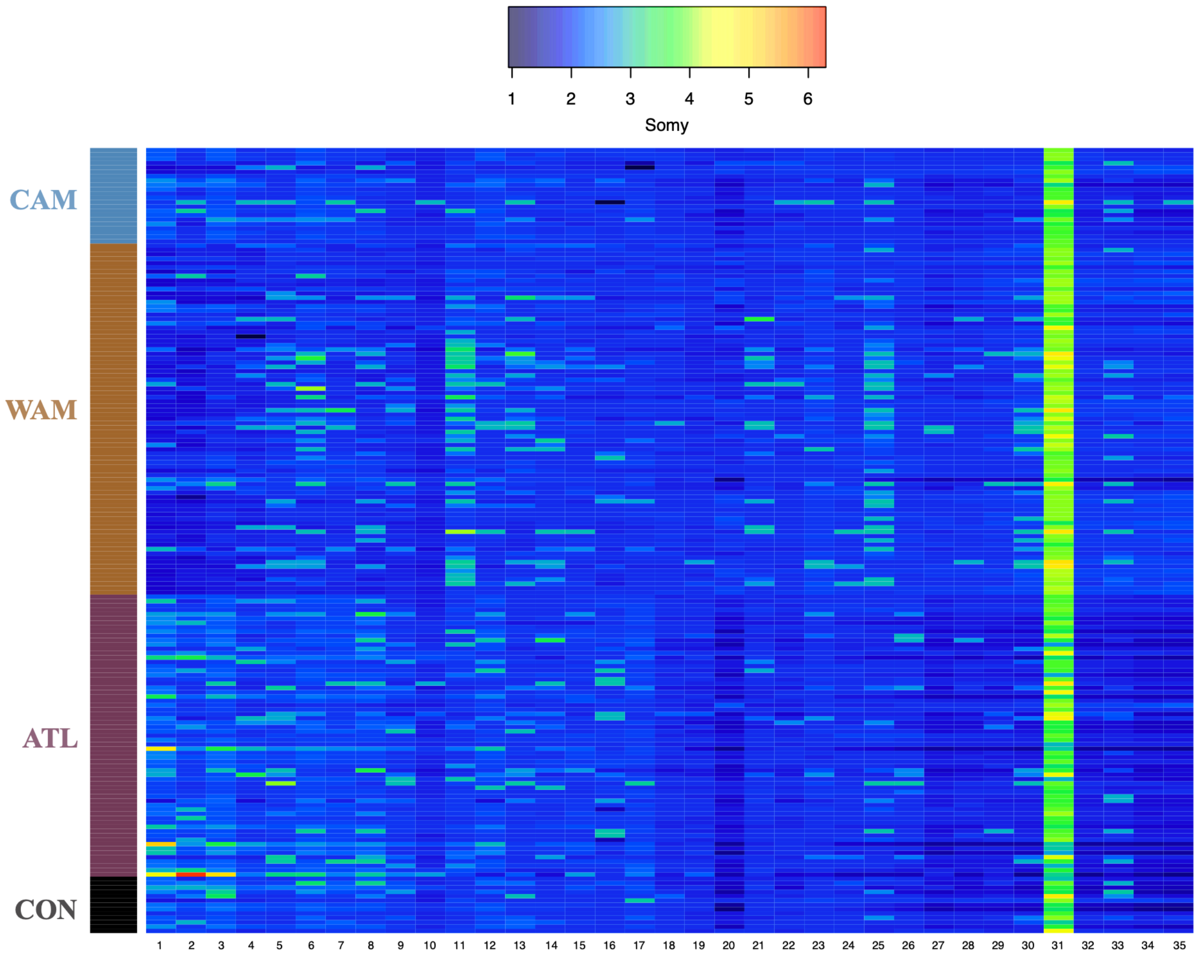

Supplementary Figure 3 Variation in chromosome copy numbers across 182 *L. braziliensis* L1 genomes. Each row in the heatmap corresponds to an isolate, which were clustered per major parasite population as inferred by ADMIXTURE and fineSTRUCTURE. CAM= Central Amazon; WAM= West Amazon; ATL= Atlantic; CON= conglomerate.

33

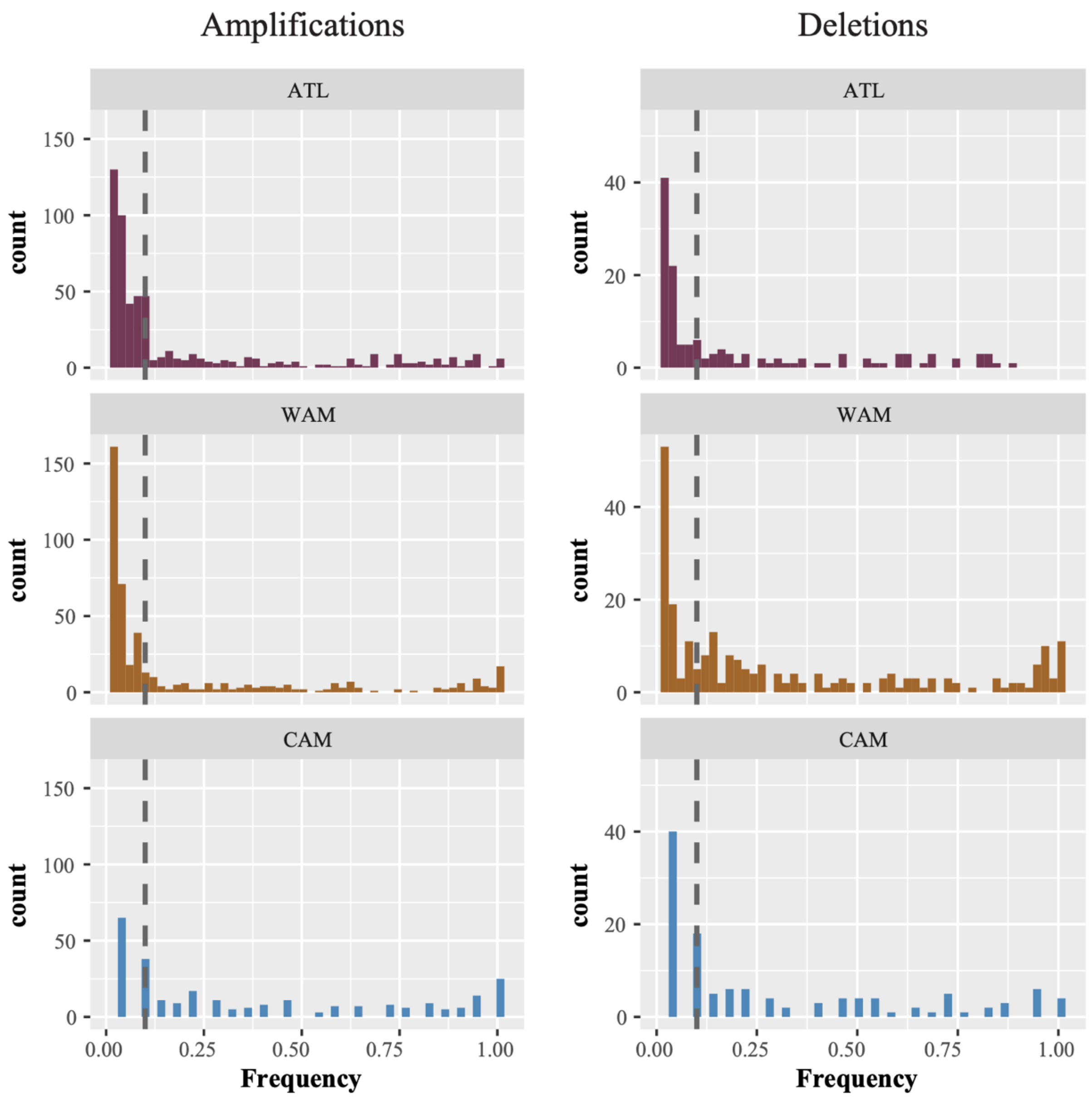

34 **Supplementary Figure 4** Frequency distribution of amplifications occurring in the three major L1 populations. (left) Amplifications. (right) deletions.

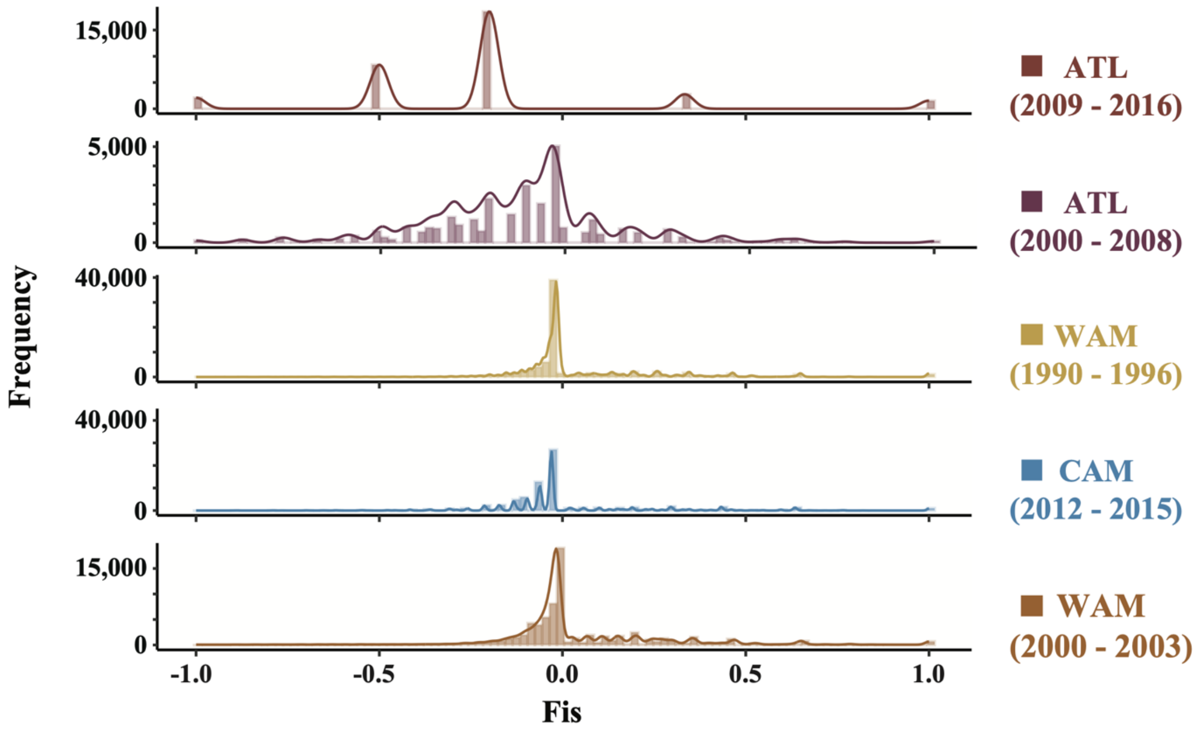

Supplementary Figure 5 Inbreeding coefficient (Fis) distributions for each of the major L1 populations, taking into account spatio-temporal Wahlund effects.

35

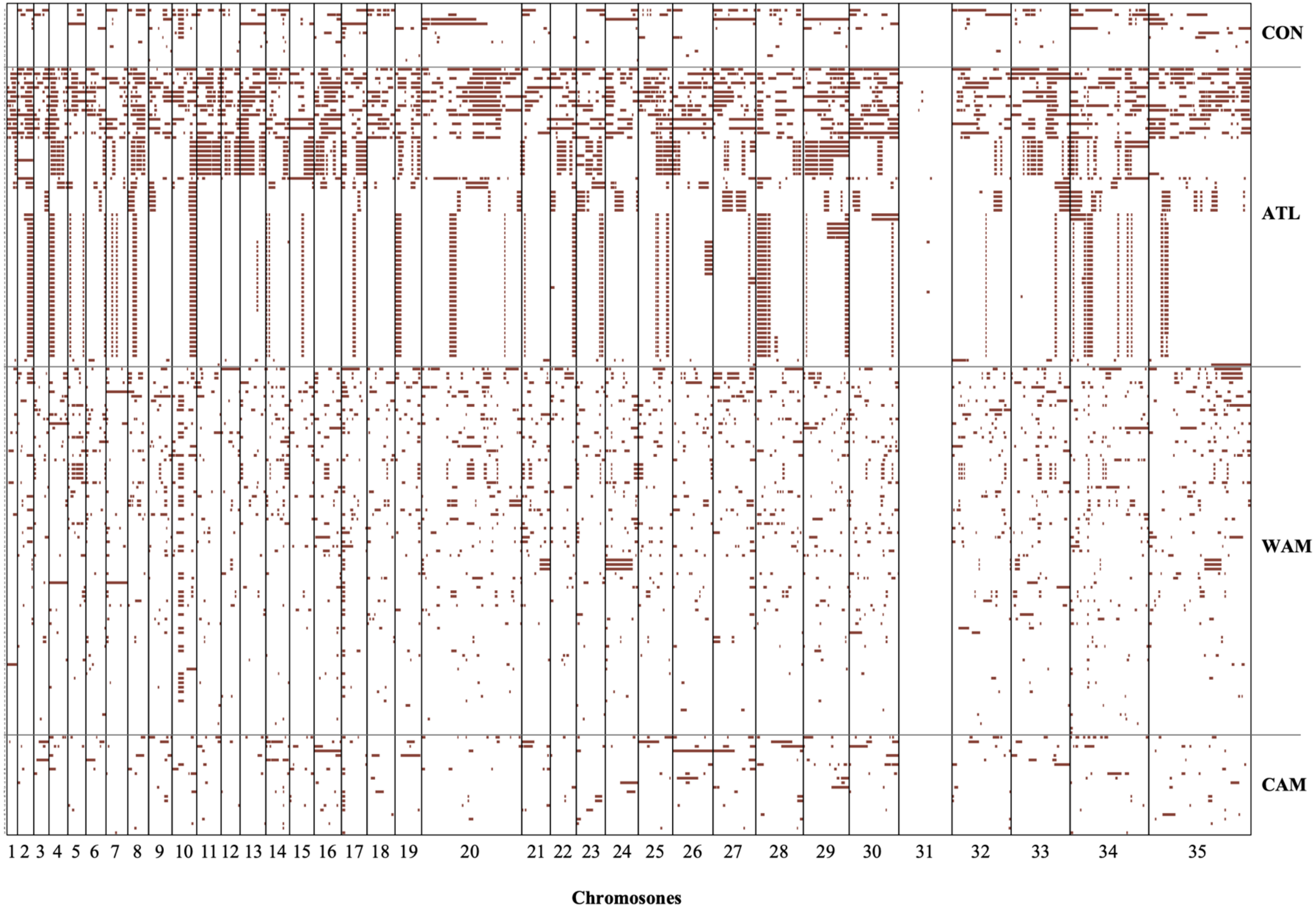

Supplementary Figure 6 Loss-of-heterozygosity pattern distribution across the genome in all of the 244 isolates.

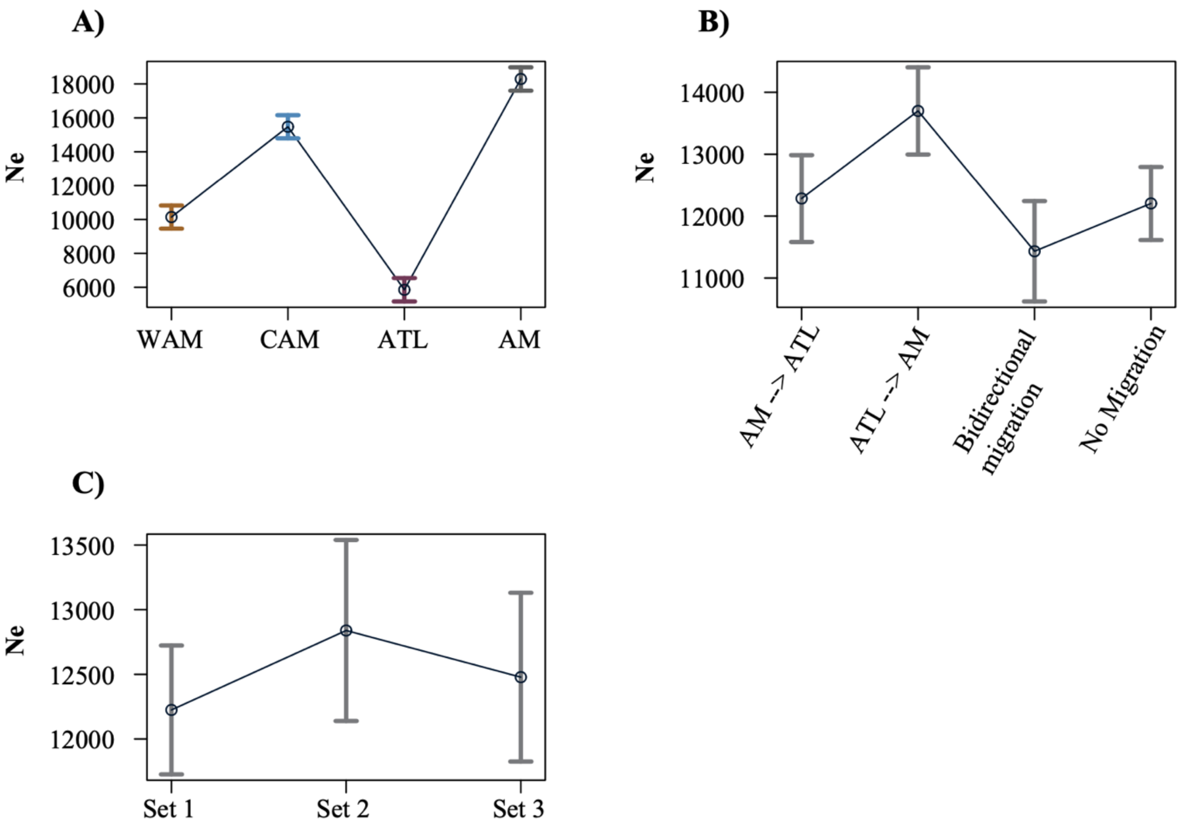

Supplementary Figure 7 Multi-way ANOVA main effect plots for Ne between the different L1 populations **(A),** migration model **(B)** and independent sample subsets **(C)**. Error bars represent the 95% confidence interval of the mean.

37

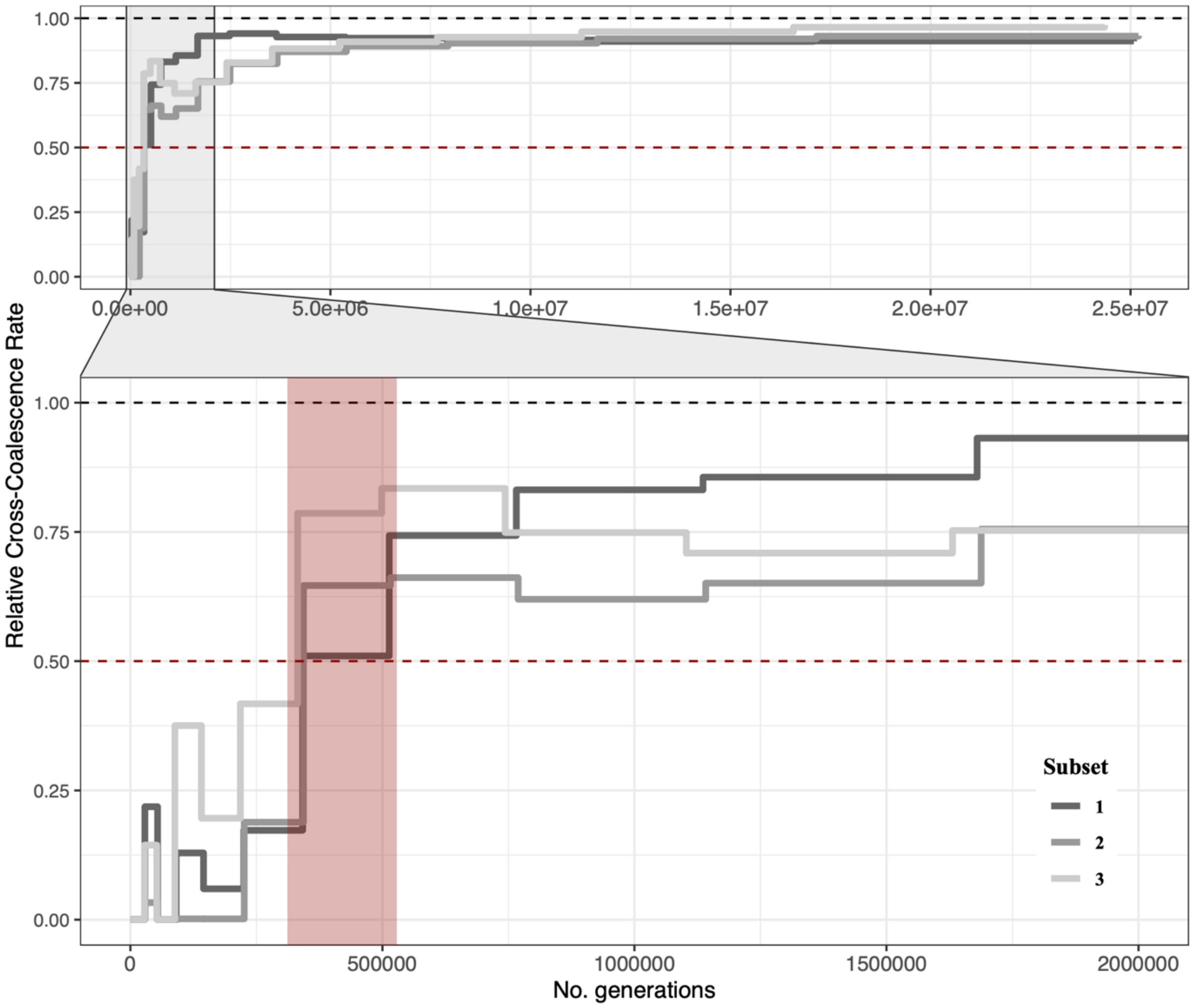

Supplementary Figure 8 Relative cross-coalescence rate (rCCR) between the two Amazonian populations (WAM, CAM). Gray lines indicate the three independent runs with different sample subsets. The red box indicates the period where the rCCR first fell to 0.5, a proposed estimate for the split time between the two populations (2).

38

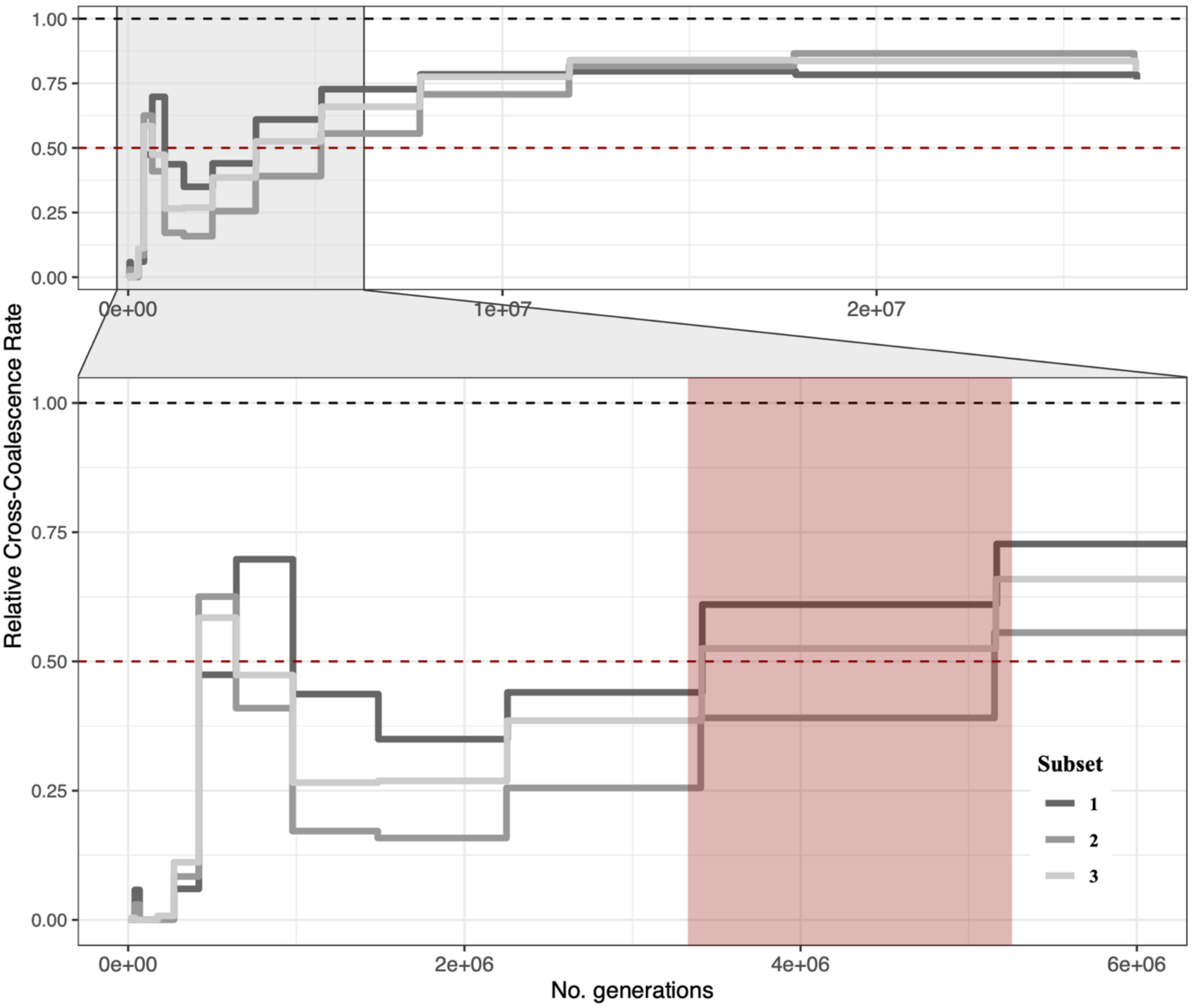

Supplementary Figure 9 Relative cross-coalescence rate (rCCR) between the Amazonian (WAM, CAM) and Atlantic (ATL) populations. Gray lines indicate the three independent runs with different sample subsets. The red box indicates the period where the rCCR first fell to 0.5, a proposed estimate for the split time between the two populations (2).

39

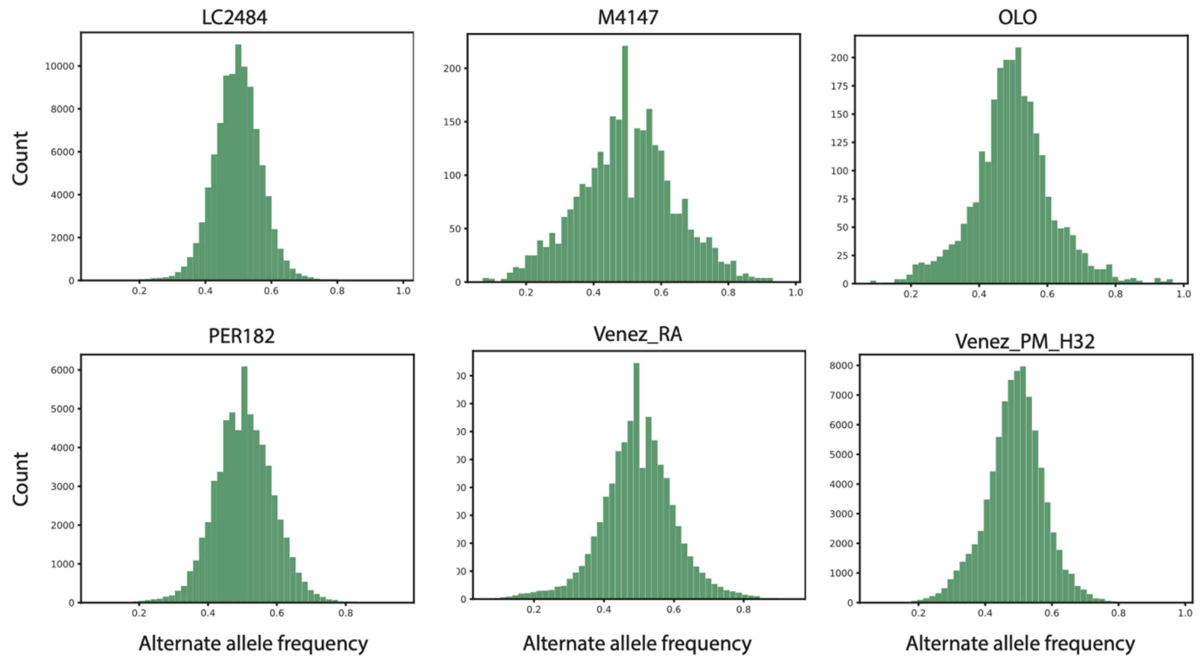

Supplementary Figure 10 Alternate allele frequency distribution at heterozygous sites for putative hybrid isolates. Each of the isolates show the expected unimodal distribution around 0.5 assuming diploidy.

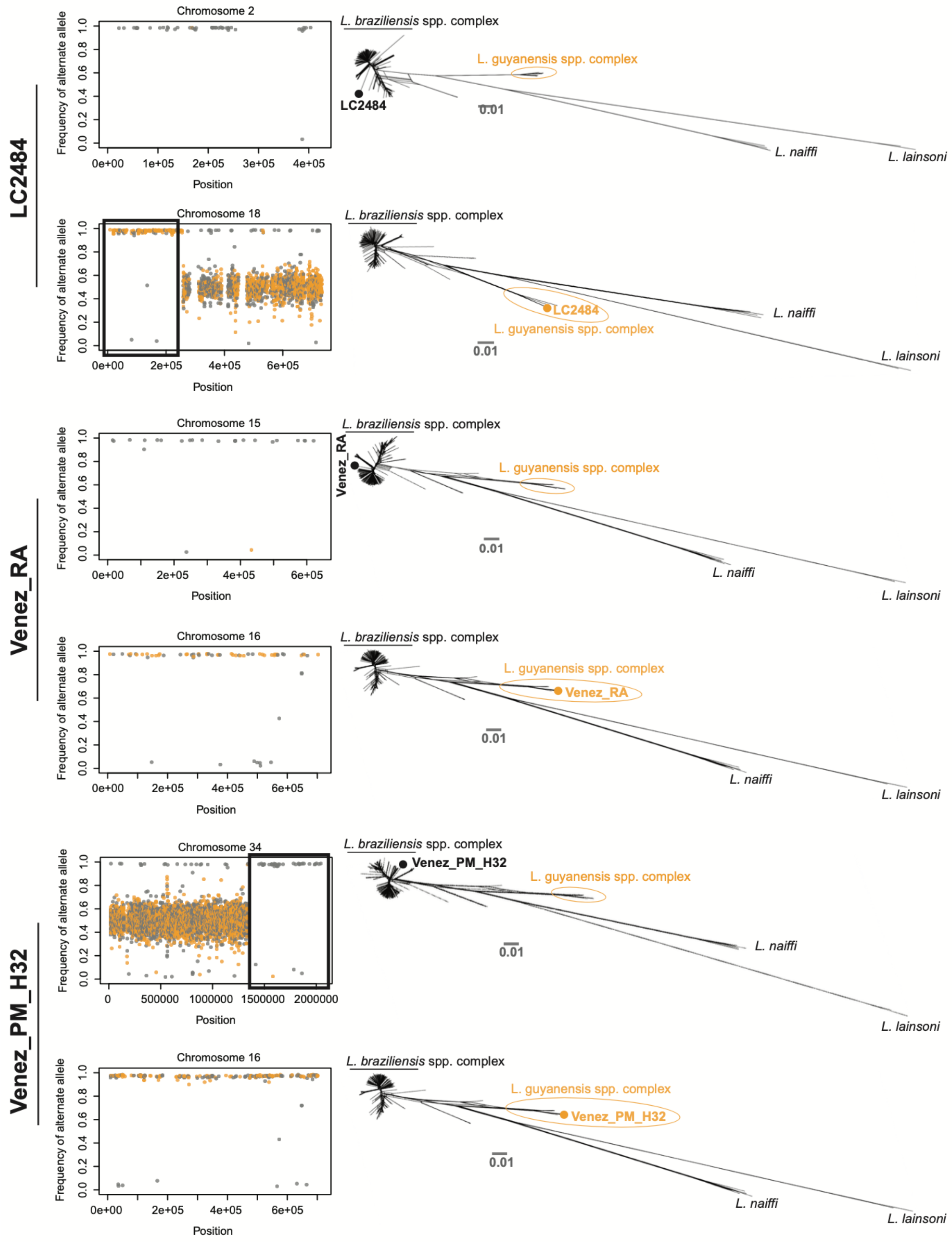

Supplementary Figure 11 Interspecific hybrids between *L. braziliensis* and *L. guyanensis*. (Left) chromosome specific frequency distributions of the alternate allele that are partially or almost entirely homozygous. Orange dots represent SNPs that are unique for *L. guyanensis*. (Right) Phylogenetic networks based on SNPs called in the presented chromosome or boxed regions (i.e. homozygous regions).

41

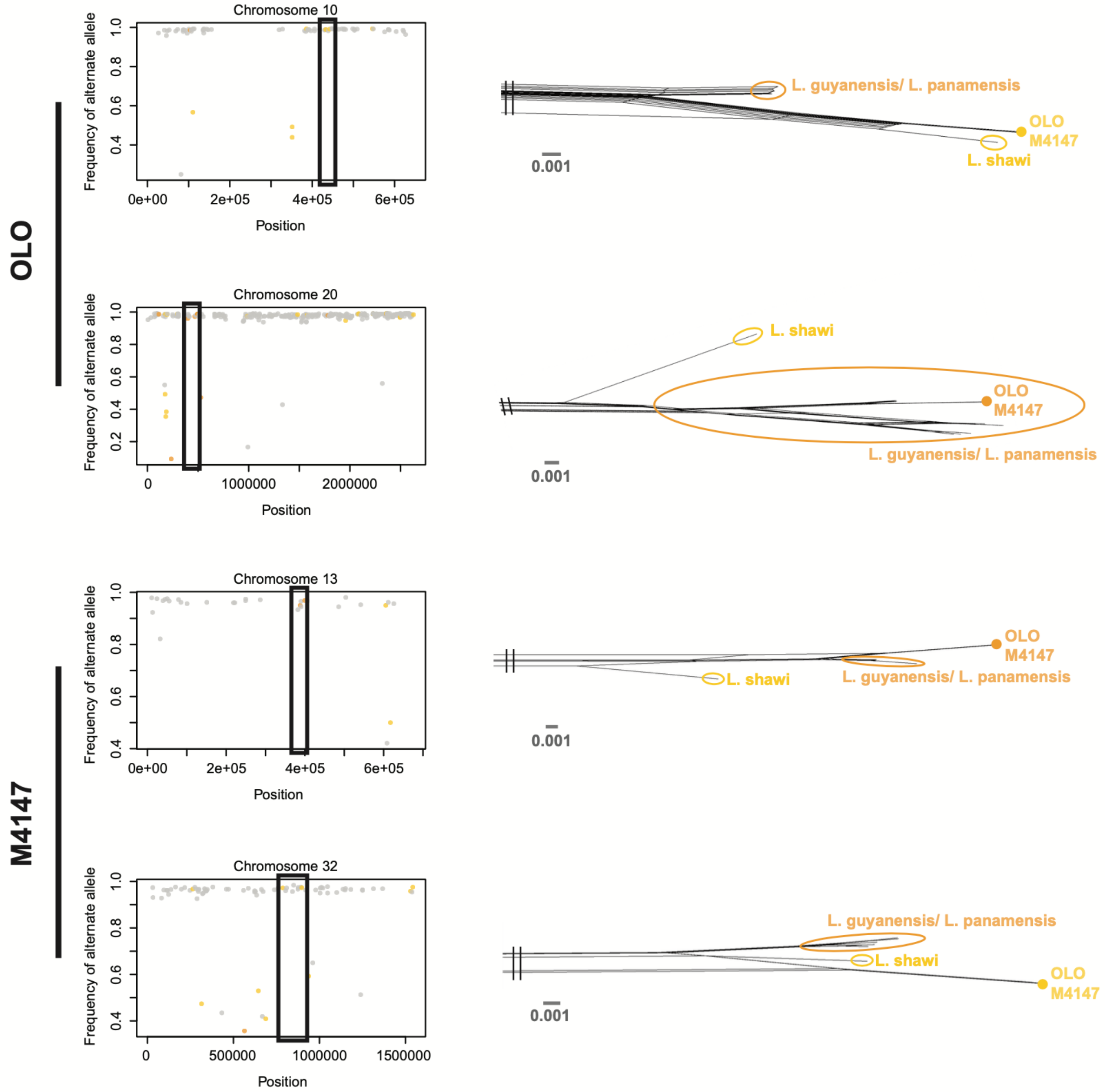

Supplementary Figure 12 Intraspecific hybrids within the *L. guyanensis* species complex. (Left) chromosome specific frequency distributions of the alternate allele that are partially or almost entirely homozygous. Orange dots represent SNPs that are unique for *L. guyanensis/L. panamensis*, yellow dots represent SNPs unique to *L. shawi*. (Right) Phylogenetic networks based on SNPs called in the presented chromosome or boxed regions (i.e. homozygous regions).

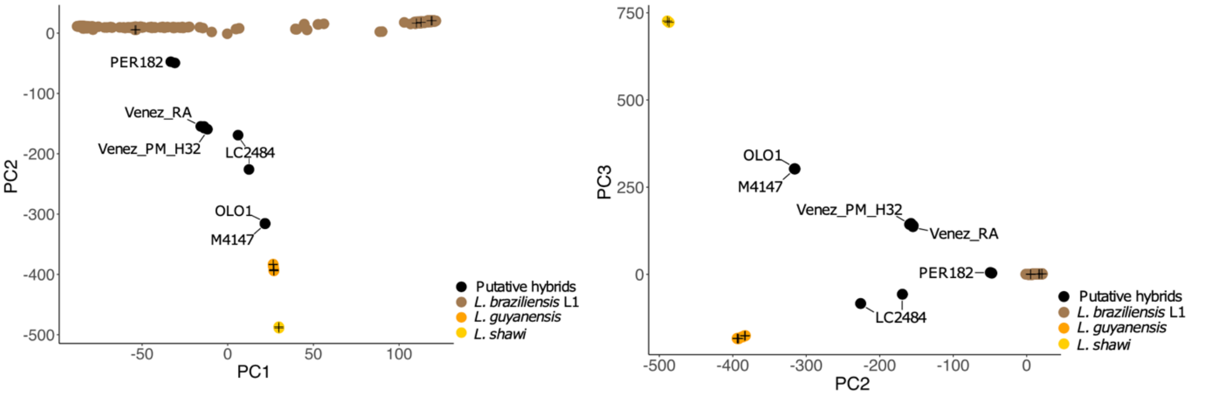

Supplementary Figure 13 PCA-based ancestry estimation of putative hybrid isolates assuming three putative parental lineages: *L. braziliensis* L1 (brown), *L. guyanensis/L. panamensis* (orange) and *L. shawi* (yellow). Plus-signs indicate isolates of the different ancestral lineages (three per lineage) that were used as controls.

References

1. Coughlan, S. *et al.* Leishmania naiffi and Leishmania guyanensis reference genomes highlight genome structure and gene evolution in the Viannia subgenus. *R Soc Open Sci* **5**, 172212 (2018).

2. Schiffels, S. & Wang, K. MSMC and MSMC2: The Multiple Sequentially Markovian Coalescent. *Methods Mol. Biol.* **2090**, 147–166 (2020).
