# Supplementary material for: Evolutionary genomics of Leishmania braziliensis across the neotropical realm
Source: Commun Biol. 2024 Nov 28;7:1587. doi: 10.1038/s42003-024-07278-z (PMC11605123; doi:10.1038/s42003-024-07278-z)
Supplement: Supplementary file 2 — Description of Additional Supplementary Files [file 42003_2024_7278_MOESM2_ESM.pdf]

Description of Additional Supplementary Files

File name: Supplementary data 1

Description: All supplementary tables associated to the manuscript.

File name: Supplementary data 2

Description: All available source data associated to the figures of the manuscript.
