# Supplementary material for: Evolutionary genomics of Leishmania braziliensis across the neotropical realm
Source: Commun Biol. 2024 Nov 28;7:1587. doi: 10.1038/s42003-024-07278-z (PMC11605123; doi:10.1038/s42003-024-07278-z)
Supplement: Supplementary file 5 — Reporting summary [file 42003_2024_7278_MOESM5_ESM.pdf]

nature portfolio

Corresponding author(s): Senne Heeren

Last updated by author(s): Nov 6, 2024

Reporting Summary

Nature Portfolio wishes to improve the reproducibility of the work that we publish. This form provides structure for consistency and transparency in reporting. For further information on Nature Portfolio policies, see our Editorial Policies and the Editorial Policy Checklist.

Statistics

For all statistical analyses, confirm that the following items are present in the figure legend, table legend, main text, or Methods section.

n/a	Confirmed	
☐	☒	The exact sample size (*n*) for each experimental group/condition, given as a discrete number and unit of measurement
☒	☐	A statement on whether measurements were taken from distinct samples or whether the same sample was measured repeatedly
☐	☒	The statistical test(s) used AND whether they are one- or two-sided<br>*Only common tests should be described solely by name; describe more complex techniques in the Methods section.*
☐	☒	A description of all covariates tested
☐	☒	A description of any assumptions or corrections, such as tests of normality and adjustment for multiple comparisons
☐	☒	A full description of the statistical parameters including central tendency (e.g. means) or other basic estimates (e.g. regression coefficient) AND variation (e.g. standard deviation) or associated estimates of uncertainty (e.g. confidence intervals)
☐	☒	For null hypothesis testing, the test statistic (e.g. *F*, *t*, *r*) with confidence intervals, effect sizes, degrees of freedom and *P* value noted<br>*Give P values as exact values whenever suitable.*
☐	☒	For Bayesian analysis, information on the choice of priors and Markov chain Monte Carlo settings
☒	☐	For hierarchical and complex designs, identification of the appropriate level for tests and full reporting of outcomes
☐	☒	Estimates of effect sizes (e.g. Cohen's *d*, Pearson's *r*), indicating how they were calculated

Our web collection on statistics for biologists contains articles on many of the points above.

Software and code

Policy information about availability of computer code

Data collection

No software was used for data collection.

Data analysis

Specific software used for data analysis:
Mapping of sequencing reads: SMALT v.0.7.4
Genome-wide variant detection (SNPs & INDELs): GATK v.4.0.2
Genomic data handling/conversion: BEDOPS v.2.4.40
calculation of alternate allele read depth frequency: vcf2freq.py script (available at: github.com/FreBio/mytools)
per-site read depth extraction: SAMtools v.1.12
Phylogenetic NeighbourNet networks: SplitsTree v.4.17.0
PCA-based ancestry estimation of the L. braziliensis species complex: PCAdmix v.1.0
Fst-calculation on per-site basis: VCFtools v.0.1.13
Model-based ancestry estimation and more in-depth population structure: ADMIXTURE v.1.3.0 & fineSTRUCTURE v.4.1.1
LD-pruning of SNPs: PLINK v.1.9
Phasing of SNPs: BEAGLE v.5.2
Calculation of LD-decay: PopLDdecay
Estimations of effective population size: G-PhoCS v.1.3.2
VCF to G-PhoCS input file conversion: vcf_to_gphocs.py script from the PPP (Popgen Pipeline Platform), available at: https://github.com/jaredgk/PPP/blob/master/pgpipe/
Estimations of effective population size through time: MSMC-2 , msmc-tools (https://github.com/stschiff/msmc-tools), SNPable (http://lh3lh3.users.sourceforge.net/snpable.shtml)
Variant annotation: SNPEFF v.5.2

R-Packages used for data analysis:
R-version: 4.0.5
RStudio: v.2023.03.01
Packages:
Principle Component Analysis: Adegenet v.2.1.7, stats package (base-R)
Pairwise Dunn's tests and Benjamini-Hochberg p-value correction: FSA v.0.9.4
Calculation of Bray-Curtis dissimilarity, Wilk's Lambda test (MANOVA): Vegan v.2.6-2
Kruskal-Wallis, Chi-squared tests, one-way ANOVA, , Tukey's HSD & main effects multi-way ANOVA: stats package (base-R)
Calculation and assessment of effective sample size (ESS) (for G-PhoCS analyses): Tracerer v.2.2.3
Survival analysis: Survival v.3.3-1; Survminer v.0.4.9

For manuscripts utilizing custom algorithms or software that are central to the research but not yet described in published literature, software must be made available to editors and reviewers. We strongly encourage code deposition in a community repository (e.g. GitHub). See the Nature Portfolio guidelines for submitting code & software for further information.

Data

Policy information about availability of data

All manuscripts must include a data availability statement. This statement should provide the following information, where applicable:

- Accession codes, unique identifiers, or web links for publicly available datasets
- A description of any restrictions on data availability
- For clinical datasets or third party data, please ensure that the statement adheres to our policy

The study was based on 257 Leishmania isolates. The metadata that was mainly used was the collection site/location, which is available as Supplementary Table 1 in the manuscript. Sequence data of all sequenced genomes usind in this study are available at Sequence Read Archive (SRA) BioProject PRJEB4442 (available as: https://www.ncbi.nlm.nih.gov/bioproject/PRJEB4442/).

Research involving human participants, their data, or biological material

Policy information about studies with human participants or human data. See also policy information about sex, gender (identity/presentation), and sexual orientation and race, ethnicity and racism.

Reporting on sex and gender	NA
Reporting on race, ethnicity, or other socially relevant groupings	NA
Population characteristics	NA
Recruitment	NA
Ethics oversight	NA

Note that full information on the approval of the study protocol must also be provided in the manuscript.

Field-specific reporting

Please select the one below that is the best fit for your research. If you are not sure, read the appropriate sections before making your selection.

☐ Life sciences ☐ Behavioural & social sciences ☒ Ecological, evolutionary & environmental sciences

For a reference copy of the document with all sections, see nature.com/documents/nr-reporting-summary-flat.pdf

Ecological, evolutionary & environmental sciences study design

All studies must disclose on these points even when the disclosure is negative.

Study description	The population genomic study was based on whole-genome data from 257 cryo-preserved Leishmania isolates, sampled over seven South American countries (Argentina, Bolivia, Brazil, Colombia, Panama, Peru and Venezuela) between 1975 and 2016. No quantitative data was obtained for this study.
Research sample	All parasite isolates were collected in light of previous studies on the genetics and epidemiology of leishmaniasis across South America. Samples were obtained from human patients with (muco-) cutaneous leishmaniasis lesions which presented themselves at local health facilities for diagnosis and treatment. Parasites were isolated from these patients for cryo-preservation. Data collections for these previous studies were ethically cleared by the ethical committees of the involved partners in the respective countries as well as the Institutional Review Board of ITM (Intsitute of Tropical Medicine, Antwerp). Within the context of this study, working with cultivated parasite isolates, no additional ethical clearance was required.

Sampling strategy	The sampling procedure at the time involved obtaining samples from leishmaniasis patients, and the number of samples depended on the number of patients presenting at health facilities in the respective countries of origin. There was no predetermined sample size: the rationale was to capture as much as possible the diversity of Leishmania parasites on a large geographic scale.
Data collection	The collection of parasites at the time were done by our partners in Argentina, Bolivia, Brazil and Peru (including Jorge Arevalo, Jorge D. Marco, Alejandro Llanos-Cuentas , Sinval Pinto Brandão-Filho and Elisa Cupolillo) and trained nurses. The recording of the sample ID, sampling location and time was done by the field staff and trained nurses.<br>Cryo-preserved parasites were grown in vitro on blood medium in order to obtain nucleic acids for sequencing, which was done by I. Maes at ITM (Antwerp, Belgium), J.D. Marco at UNS (Salta, Argentina) or M.C. Boité, L.M.Cantanhêde and K. Chourabi at Fiocruz (Rio de Janeiro, Brazil). Sequencing was done by M. Sanders at the Welcome Sanger Institute.
Timing and spatial scale	Samples were obtained between 1975 and 2016 from at least 79 different localities in seven South American countries (Argentina, Bolivia, Brazil, Colombia, Panama, Peru and Venezuela). These samples were collected during multiple studies investigating the genetics and epidemiology of leishmaniasis in the different regions. The exact location and timing of sampling, together with the latitude and longitude, can be found in Supplementary Table 1, as described in the paper.
Data exclusions	All data were included for sequencing. Thirteen parasite genomes were subsequently removed for downstream analyses either because they showed i) aberrant allele read depth frequencies, or ii) a low or fragmented coverage of the accessible genome (as described in the manuscript).
Reproducibility	All results were based on describing the parasite's genomic diversity using a set of parasite isolates that were collected during previous studies. The concordance in results within the study (as obtained from different analyses) and with previous work (e.g. Heeren et al. 2023; Van den Broeck et al. 2020) reflect the reproducibility of our study. Results can be reproduced by following the detailed methods section.
Randomization	As the main purpose of this study was to obtain the entire extent of the genomic diversity of Leishmania braziliensis as much as possible, randomization was not applicable as all isolates were subjected to DNA extraction and sequencing.
Blinding	There was no blinding to data acquisition nor data analysis. Blinding was not relevant to this study.

Did the study involve field work? ☐ Yes ☒ No

Reporting for specific materials, systems and methods

We require information from authors about some types of materials, experimental systems and methods used in many studies. Here, indicate whether each material, system or method listed is relevant to your study. If you are not sure if a list item applies to your research, read the appropriate section before selecting a response.

Materials & experimental systems

n/a	Involved in the study
☒	☐ Antibodies
☒	☐ Eukaryotic cell lines
☒	☐ Palaeontology and archaeology
☒	☐ Animals and other organisms
☒	☐ Clinical data
☒	☐ Dual use research of concern
☒	☐ Plants

Methods

n/a	Involved in the study
☒	☐ ChIP-seq
☒	☐ Flow cytometry
☒	☐ MRI-based neuroimaging

Plants

Seed stocks	NA
Novel plant genotypes	NA
Authentication	NA
